# Academic help-seeking behaviour and barriers among college nursing students

**DOI:** 10.4102/hsag.v29i0.2425

**Published:** 2024-01-18

**Authors:** Million S. Bimerew, John P. Arendse

**Affiliations:** 1 School of Nursing, Faculty of Community and Health Sciences, University of the Western Cape, Cape Town, South Africa

**Keywords:** academic expectation, academic performance, academic workload, academic support, college nursing students, help-seeking, perceived stress, help-seeking as a threat to self-esteem

## Abstract

**Background:**

First-year college student’s smooth transition and academic success influenced by academic help-seeking behaviour. Academic help-seeking behaviour is largely affected by many factors, including demographic factors, self-esteem and the use of sources for academic learning.

**Aim:**

The study investigated academic help-seeking behaviour and barriers among first-year college nursing students.

**Setting:**

The study was conducted at a nursing college in the Western Cape province of South Africa.

**Methods:**

A cross-sectional descriptive survey design with a self-administered questionnaire was used to collect data from 130 first year nursing college students. Descriptive statistics and bivariate analysis were computed using Statistical Packages for Social Sciences (SPSS).

**Results:**

More than 77.7% used course materials and books to help with academic learning, 50% of students sought help from their teachers. Only 24.6% and 17.7% of students used YouTube and computers respectively. In all items measured help-seeking is not a threat to self-esteem, teachers and parents did not have unrealistic expectations of their academic performance. Language is significantly associated with (*p* < 0.001) academic help-seeking behaviour.

**Conclusion:**

Most students mainly used informal sources for academic learning. Help-seeking was not a threat to self-esteem. The language barrier is significantly associated with academic help-seeking behaviour. The nursing college should provide a coordinated academic language support, academic consultation and counselling services for academically stressed first-year nursing students.

**Contribution:**

The findings highlighted language as a barrier to academic help-seeking. The study provides insight to strengthen the language and academic support for academic learning for first year nursing students.

## Introduction

Transitioning from high school to college is not easy for first-year students, including adjustment problems to the new life situation away from the primary family support structure, social network, demanding academic work and emotional stress (Gosai, Tuibequ & Prasad 2023; Van Rooij et al., 2018). It is documented that transitioning from a high school basic education environment to a college education environment is stressful (Van Rooij et al. 2018). According to Van Rooij, Jansen and Va de Grift (2018), the issue of poor academic adjustment among first-year undergraduate students is caused by both internal and external factors that have an impact on academic performance and persistence intentions.

A survey study identified that 64.1% of students found developing close friendships with their peers to be very challenging and this made it difficult for them to seek academic help from friends (Tran, Williams & Yaji 2022). An older study conducted among 194 first-year university students in South Africa identified that 59% of the students had adjustment problems because of psychosocial factors (Petersen, Louw & Dumont 2009). Despite numerous factors affecting first-year students transitioning into a higher education environment, academic help-seeking behaviours help for smooth transitioning and increase academic success in terms of achievement (Rienties & Tempelaar 2012).

One method students may employ to overcome obstacles and succeed academically is academic help-seeking behaviour (Deng et al. 2022; Ratanasiripong et al. 2020). Students can utilise a variety of academic help strategies to increase their learning, such as organising their studying, participating in their coursework and stress management. Academic help-seeking is the process of realising one needs help, deciding what one does, finding helpers, soliciting aid and assessing the service (Van het Onderwijs 2016). Students must communicate and learn socially to seek help for academic learning. Depending on a student’s gender, race or academic standing, different help-seeking behaviours will emerge (Fong et al. 2021). According to Umubyey et al. (2016), the fundamental idea of academic help-seeking entails a person who has a need that can be satisfied with the support of others. There are two types of help-seeking behaviour among students, which are formal and informal (Makara & Karabenick 2013). Formal help-seeking behaviour by students refers to getting support from a qualified source such as a teacher, tutor, support service or online class portal (Makara & Karabenick 2013). Contrarily, informal help-seeking behaviour uses unofficial sources such as friends, family, the internet and other people (Shumet et al. 2021). Informal help-seeking may be less stressful but may not produce high-quality information, whereas formal help-seeking may be more uncomfortable but will produce better results (Shumet et al. 2021). Help-seeking in academic learning was positively related to academic performance while avoiding asking for help was negatively correlated with academic performance (Deng et al. 2022).

Academic help-seeking behaviours are also largely influenced by the perceived threat to one’s self-esteem and language skills (Balwant 2018; Lawson & Lawson 2020). Studies on characteristics associated with students, such as self-worth, show that students who feel lower self-esteem ask for assistance, whereas students who feel insecure about their knowledge or language skills in a course were less likely to ask questions or academic assistance (Deng et al. 2022; Qayyum 2018). Evidence suggests that self-esteem affects interpersonal interactions, academic success and mental health (Jang & Han 2020; Kassahun & Alemihun 2022); self-esteem is also directly associated with the behaviour of academic help-seeking (Chunhua, Yongfeng & Youpeng 2022; Xueying 2022). Research evidence has shown that those who are self-regulated learners and aware of their shortcomings have a unique perspective on learning, while those who do not like to ask for help when they need it are demonstrating disengagement from the learning process, which may impede their capacity to learn and academic performance (Balwant 2018; Lawson & Lawson 2020). Furthermore, students in traditional face-to-face classes felt more intimidated to seek help than those enrolled in distance education (DE) courses (Qayyum 2018). In terms of self-esteem, an older study suggests that a variety of personality qualities, including self-respect, locus of control, shyness and authoritarianism, autonomy, introversion, dependence, conservatism, rigidity and individualism, might have an impact on one’s sense of self-worth (Aphroditi & Zartaloudi 2010). Students who lack confidence or have low self-esteem are reluctant to engage in learning or take necessary risks for their academic advancement (Kassahun & Alemihun 2022).

On the other hand, students’ age and gender have an impact on students’ help-seeking behaviour, for instance, younger students may feel threatened about whether asking for help is acceptable and suitable (Bosworth & Kersley 2015). Male students are less likely than female students to ask for academic assistance, and they often perform worse academically than female students (Marrs, Sigler & Brammer 2015). Therefore, low self-esteem and demographics are barriers to help-seeking behaviour.

There seems to be a general agreement among scholars that understanding the language of instruction is among the factors that could play substantial roles in students’ academic success (Civan & Coşkun 2016). Brown, Barry and Todd (2020) found that students’ help-seeking behaviours are greatly impacted if they are using a second language as a medium of instruction in the classroom. Students with weak language skills feel that they cannot participate in classroom activities or extracurricular activities. It is emphasised that these students will never be able to participate in classroom discussions or academic learning. Conversely, students with strong language skills can confidently participate in classroom discussions, ask questions and engage in extracurricular activities. Students with lower levels of English language proficiency are also less likely to seek academic assistance (Brown et al. 2020). A substantial body of research has been done on the subject, and the consensus is that when students receive instruction in a language other than their mother tongue, it has a detrimental impact on their desire to seek academic support (Civan & Coşkun 2016). According to a Turkish study, instruction in English reduces students’ academic engagement and success, particularly in their first or freshman year (Owings, Kaplan & Pirim 2012). Bernhofer and Tonin (2022) found that completing a test in a second language results in a 9.5% grade point loss. Because English is the primary language of teaching in higher education in South Africa, second-language learners find it extremely difficult to participate in class and seek out academic support (Prinsloo, Rogers & Harvey 2018). It is therefore important to understand the impact of language barriers on student’s academic help-seeking behaviour and self-esteem.

According to research, the majority of students (70%) sought assistance from at least one informal source, such as classmates, and at least one professional source, such as academic student services (62%). Qayyum (2018) found that students are more likely to seek assistance from classmates or other students (63%) than from teachers (47%). Students’ desire to work independently and their perception of help-seeking are important predictors of whether they ask teachers for help outside of class (Mahasneh, Sowan & Nassar 2012). Moreover, in a study by Koc and Liu (2016), more than half (53.8%) of students preferred informal sources, such as contacting classmates and using email for help-seeking, and also one-third of students posted a question in the class discussion board. Studies on undergraduate college students indicate that when students are faced with academic challenges, they seek assistance mainly from informal sources to resolve the challenges, which include friends, peers, family, classmates, other instructors, consult books and information seeking from an information system (Algharaibeh 2020; Giblin 2016; Thomas & Tagler 2019). A study on an online course identified that students preferred using digital technologies to seek help from both instructors and fellow students (Koc & Liu 2016).

It is during this transition period that first‑year students face many challenges, including moving away from their primary family support systems and more demanding coursework. To this end, some studies have suggested that first-year students need a support structure including how to seek academic help from senior students and social networking (Algharaibeh 2020). The researcher of this study had his own experience of teaching first‑year students and had observed students’ disengagement academically and socially, which led to poor academic performance. Firstly, the academic reports show that students’ academic achievement record was frequently daunting and had high failure rate, which was concerning for the course instructor. Secondly, in the college, while there are different types of research studies conducted on different issues, the researcher did not come across such a study on the topic area. The researcher was interested in finding out the reasons for students’ disengagement and poor academic performance, and the results would help him to develop an intervention to support those students who need academic support. Hence, this study examined academic help-seeking behaviours and barriers to help-seeking behaviour among first-year college nursing students in the Western Cape.

### Aim

The study investigated academic help-seeking behaviours and barriers among first-year college nursing students.

### Objectives

The objectives of this study were:

- To describe the academic help-seeking behaviours of first-year college nursing students.
- To determine help-seeking as a threat to self-esteem among first-year students.
- To determine the demographic characteristics that influence academic help-seeking behaviour.
- To describe the sources of academic help among first-year college nursing students.

## Research methods and design

### Study design

A cross-sectional descriptive research design was employed to conduct the study. A self-administered questionnaire was used to collect data from all first-year college nursing students who participated voluntarily in the study.

### Study setting

This study took place at a nursing college in the Western Cape, South Africa. The nursing college provides a four-year nursing diploma programme. The college was purposively selected because it is one of the largest nursing colleges in the province, which recruits students from other cities and provinces in the rule areas. These students coming particularly from rural areas have difficulties in adjusting to big city life and academic environment, including language problems. Thurber and Walton (2012) indicated that separation from home can cause homesickness, which is distressing and in turn might reduce academic success, and this is true, particularly with first-year college nursing students, hence the selection of the study setting.

### Population and sampling strategy

In this study, the population was all first-year college nursing students registered in 2019 for the diploma programme. The total number of first-year nursing students who formed the study population was 170. Sampling is a process of selecting a portion or subject of the designated population to represent the entire population, which enables to generalise the population from which the sample was drawn (Creswell 2014:170). In this study, the total number of first-year nursing students’ size was small; therefore, a census sampling technique was used to include all first-year nursing students in the study. The inclusion criteria were all first-year nursing students registered for diploma programmes in 2019, and the exclusion criteria were students less than 18 years at the time of data collection.

### Data collection instrument

‘Psychosocial factors that predict the college adjustment of first-year undergraduate students: Implications for college counselors’ questionnaire developed by Martin, Swartz-Kulstad and Madson (2011) was adapted after a written permission was obtained from the original authors. The questionnaire was written in the English language, which was the language of the academic medium of the respondents of this study. Minor modification of the questionnaire was made by changing some naming and wording to the context of the study. The questionnaire consisted of a Likert-type rating scale. The rating scale comprised three options: (1) for disagree; (2) for uncertain and (3) for agree. The questionnaire was classified into five sections, namely: (1) demographic data, (2) sources of help-seeking, (3) help-seeking in understanding the subject, (4) help-seeking as a threat to self-esteem and (5) perceived stress related to academic expectation. In respect of seeking help as a threat to self-esteem, all the statements were negatively constructed – seeking help is a threat to self-esteem.

### Data collection

All the first-year class students were invited to participate in the study. The purpose of the study was explained to the respondents, and they were informed that participation in the study was voluntary; they had the right not to participate at all or the right to withdraw from participating in the study at any time without any consequences. They were also informed that the anonymity and confidentiality of the information they provided would be maintained during and beyond the study period. The respondents were informed that there was no need to write their names or any identification on the questionnaire. After informed consent was obtained from those who agreed to participate, the questionnaires were distributed by one of the lecturers who was not teaching the first-year programme. The researcher was present during the data collection to answer the respondents’ questions and assist them in completing the questionnaire; the presence of the researcher did not influence the students to complete the questionnaire, as 40 students showed no interest in participating in the study. About 130 completed questionnaires were returned with a response rate of 76.5%. The data were collected in November 2019.

### Data analysis

The statistical analyses were performed using IBM Statistical Packages for Social Sciences (SPSS) version 24. The summary scores for the Likert scale were calculated for each item. Descriptive statistics such as frequencies and percentages were used to present categorical data. The chi-square test was conducted to determine the association between categorical variables. The *p*-value of < 0.05 was considered statistically significant.

### Reliability and validity

The adapted tool was reviewed by content experts in the field who rated each item on the survey on a five-point scale. A content validity index (CVI) was calculated for each scale by calculating the percentage by three experts who rated the scale items as 3 (quite relevant) or 4 (highly relevant). The CVI was 100% on all scales. Cronbach’s alpha reliability coefficients for the subscales ranged from 0.70 to 0.0.91. The overall tool has adequate internal consistency, with a total calculated Cronbach’s alpha of 0.91.

### Ethical considerations

Ethics approval was obtained from the ethics the Humanities and Social Science Research Ethics Committee of the University of the Western Cape (Ref. No. HS17/10/19). Permission to undertake the study was obtained from the Director of the College of Nursing to access the respondents. Informed consent was obtained from all of the respondents before the data collection began. Confidentiality and anonymity were ensured during data collection, analysis and beyond the study period.

## Results

### Sociodemographic factors in seeking help for academic learning

The main age group of those who sought help for academic learning was under the age of 20 years, followed by the age group of 21–25 years. A statistically significant value (χ² = 21.169, *p* = 0.055) was not found for the age category regarding the use of help-seeking in academic learning. More than half of 59 (51%) single respondents did not use help-seeking as a source of learning, compared to 7 (47%) of the married respondents who reported never doing so. Marital status showed no statistical significance (χ² = 6.897, *p* = 0.075) regarding the use of help-seeking for academic learning. About 35 (69%) of the Xhosa-speaking and 19 (56%) of the Afrikaans-speaking respondents never sought help for academic learning. There was a statistically significant association (χ² = 27.076, *p* = 0.001) between home language and the use of help-seeking in academic learning (Table 1). Students with English as a home language often seek help for academic learning than Afrikaans-or Xhosa-speaking students.

**TABLE 1:** Influence of demographic factors in seeking help for academic learning.

	Never		Sometimes		Often		*p*
	*n*	%	*n*	%	*n*	%	
**Gender**							
Male	15	71	4	19	2	10	0.127
Female	51	47	21	19	37	33	
**Age group (years)**							
Under 20	35	59	7	12	17	29	0.055
21–25	22	45	9	18	18	34	
26–30	6	55	2	18	3	27	
31–35	1	33	1	33	1	33	
36 and older	2	25	6	75	0	0	
**Marital status**							
Married	7	47	5	33	3	20	0.075
Single	59	51	20	17	36	32	
**Home language**							
English	10	26	10	26	18	48	0.001
Afrikaans	19	56	8	24	7	21	
Xhosa	35	69	5	10	11	22	
Other	2	29	2	29	3	43	

### Use of sources of help available in academic learning

The study determined the use of various sources of help available for academic learning by first-year college nursing students. The findings show that about 66 (50.8%) of the respondents never used their friends as sources of help, while 56 (43%) and 49 (37.7%) of the respondents reported that they often and sometimes made use of the teachers, respectively. Most of the respondents, 101 (77.7%), often use course manuals and books. The use of computer tutorials and YouTube to help with academic learning was assessed, and the results show that 70 (53.8%) and 74 (56.9%) respondents never use computer tutorials or YouTube, respectively (Table 2).

**TABLE 2:** Use of sources to help in academic learning.

Sources of help in academic learning	Never		Sometimes		Often	
	*n*	%	*n*	%	*n*	%
Friends	66	50.8	25	19.2	39	30
Teacher (course instructor/lecturer)	25	19.2	49	37.7	56	43
Course manual and books	8	6.2	21	16.2	101	77.7
Computer tutorials	70	53.8	37	28.5	23	17.7
YouTube videos	74	56.9	24	18.5	32	24.6

### Help-seeking behaviour of students in understanding the subject matter

In measuring the academic help-seeking behaviour of students during difficulty in understanding the subject, about 64 (49.2%) respondents sought help to understand the learning material (Table 3). More than half, 69 (53.1%), of the respondents asked the teacher to go over the course material with them, and 56 (43.1%) asked the teacher to explain what they did not understand about the course matter. However, 61 (46.9%) did not get help with the parts they did not understand.

**TABLE 3:** Help-seeking behaviour of students in understanding the subject matter.

Help-seeking behaviour of students	Disagree		Uncertain		Agree	
	*n*	%	*n*	%	*n*	%
I get some help to understand the learning material better.	37	28.5	29	22.3	64	49.2
I ask the teacher to go over it with me.	19	14.7	42	32.3	69	53.1
I ask the teacher to explain what I did not understand.	32	24.6	28	21.5	70	53.9
I get some help on the parts I did not understand.	61	46.9	21	16.2	48	37

### Seeking help as a threat to self-esteem during academic learning

The findings indicated that 82 (63%) of the respondents disagreed with the statement that they stay away from people when they are not doing well in school, and 117 (90%) disagreed with the statement that they do not want to see anyone when they are not doing well in school. More than two-thirds, 108 (83.1%), of the respondents talked to anyone when they were not doing well in school, and the majority 111 (85.4%) of the respondents did not keep people away from finding out when they were not doing well in school. In general, help-seeking for academic learning is not considered a threat to self-esteem by most of the respondents (Table 4).

**TABLE 4:** Help-seeking as a threat to self-esteem during academic learning.

Help-seeking as a threat to my self-esteem, when am I not doing well in my academic learning	Disagree		Uncertain		Agree	
	*n*	%	*n*	%	*n*	%
I stay away from people.	82	63.0	25	19.2	23	17.7
I don’t want to see anyone.	117	90.0	10	7.7	3	2.3
I don’t want to talk to anyone about it.	111	85.4	6	4.6	13	10.0
I don’t want to talk about it.	108	83.1	6	4.6	16	12.3
I try to keep people from finding out.	111	85.4	12	9.2	7	5.3
I make sure nobody finds out.	116	89.2	10	7.7	4	3.0
I try to hide it.	112	86.1	8	6.2	10	7.7
I don’t tell anyone about it.	114	87.6	12	9.2	4	3.1
I don’t let anybody know about it.	113	86.9	8	6.2	9	6.9

## Discussion

We measured the academic help-seeking behaviours and barriers among college nursing students. The study examined whether the demographic factors of gender, age, home language and marital status correlate with academic help-seeking behaviours. It was identified that home language was found a greater influence on academic help-seeking behaviours. There was a significant (*p* = 0.001) association found between language and academic help-seeking behaviour, with a significant number of the Xhosa- (35, 69%) and Afrikaans-speaking (19, 56%) students perceiving language as a barrier to seeking help for academic learning. The college uses English as the medium of instruction to teach academic subjects, and students must use the English language to ask questions or for any academic assistance. The students who were predominantly Xhosa- or Afrikaans-speaking were mostly from rural areas, and they might have felt their English was not good enough to speak in front of the students who came from cities. This could also influence with whom they may become friends or avoid any friends, which may lead to academic disengagement. A similar study identified that nursing students experienced difficulty expressing themselves in writing, especially if English is their second language (Hendrix et al. 2012). The study could not find gender and marital status as a barrier to academic help-seeking behaviours. In contrast, previous studies found that demographic factors such as age and gender had affected academic help-seeking behaviour (Bornschlegl, Meldrum & Caltabiano 2020; Koydemir-Özdena & Erelb 2010). For instance, females have more positive attitudes towards psychological and social help-seeking than their counterpart, and women tend to seek out help from outside sources than males (Atik & Yaltyn 2011). Research also shows that young adolescents tend to seek help from informal sources, compared to older people who are more likely to seek help from a professional person (Hanafi & Noor 2016; Koydemir-Özdena & Erelb 2010). Further, Giblin (2016) revealed that privately searching information from books not only removes the drawback of embarrassment but also reduces the likelihood of finding a customised answer.

In determining the sources of help for academic learning, more than two-thirds (77.7%) of the students often used course materials and books, and 43% of the students got academic help from their course instructors. More than 50% of the students never used informal sources, such as friends, computers and YouTube, as a source of help for academic learning. Traditionally, students rely on the teacher’s notes and course materials provided by the institution, as this would give them more confidence for passing tests and exams. In contrast, earlier research shows that college students do not seek academic help through formal channels, particularly those students struggling academically who prefer to seek help informally. A more recent study also shows that college students are seeking academic help from informal sources, such as peers, friends and email (Fong et al. 2021). Some studies have shown that formal help-seeking is more positively associated with students’ grades (Fittrer 2016). Amemiya and Wang (2017) found that seeking help from teachers can increase academic self-concept and benefit lower-achieving students. The study also identified that students did not use online sources, such as YouTube or computer tutorials, as sources of tools for academic learning. However, in the current higher educational climate, YouTube is used as an educational tool for academic learning and improving students’ general knowledge. Higher educational institutions are supposed to prepare students for the integration of information and communication technology in their curriculum to provide a dynamic and proactive teaching and learning environment. A similar study indicated that students use fewer formal sources for academic help (Qayyum 2018), and most students rely on computer devices, social media or phones as communication devices in seeking help for academic purposes (Cilliers 2017; Qayyum 2018). The reason for the different findings in the current study might be many of the students were from rural areas, and they trust the formal sources of information obtained from their instructor for passing the subject more than the informal sources. The other possible reason could be that the students may not access online platforms, such as YouTube, or computers to use it as an academic learning tool.

Four questions were used to examine student’s academic help-seeking behaviour when having difficulty understanding the subject matter. In general, more than 50% of the students sought academic help when having difficulty understanding the subject matter. More than 53% of the students did ask their instructor to go over the subject matter or to explain the subject matter for more clarity. Although the source of help was not specified, closer to 50% of the students reported getting some help to understand parts of the subject matter when they experienced difficulty with it. These findings are in line with those of Thomas and Tagler (2019) who reported that seeking academic help can result in positive academic outcomes. On the other hand, although the universities have academic learning resources available, they are unfortunately underutilised, and students who have busy schedules and live off-campus use less of the university-based academic support systems for academic learning (Thomas & Tagler 2019). This study did not investigate the reasons for not seeking academic help; however, Osborne (2019) identified that a common reason for not seeking academic help might be the students’ fear that other people may consider them incompetent. Morales (2014) identified that students do actively seek academic help when faced with challenges. In his theory of self-efficacy, Bandura (2006) explains help-seeking behaviour as being a component of self-efficacy, which consists not only of self-regulated learning but also of one’s belief or strategy that one can perform well on a designated academic task, and aids in achieving academic success in the face of difficult or challenging tasks. In other words, help-seeking behaviour is not an indication of dependency but rather is the use of assistance from others when the need arises.

The study assessed the extent to which first-year college nursing students perceived academic help-seeking as a threat to their self-esteem. There appear to be a variety of factors that influence how students act when they seek academic help; for instance, while some aspirations for academic success and self-affirmation can promote positive help-seeking behaviours, others, such as low self-esteem, can have an effect on students’ attempts to acquire help. Low self-esteem, which usually brings reluctance to recognise their own mistakes, is the key factor contributing to students’ perception of help-seeking risks (Reeves 2015). However, in the current study, most of the students reported that academic help-seeking is not a threat to their self-esteem, which means discussing their academic problems with others is not a threat to their self-esteem. A similar study reported that college students’ help-seeking, given the prospect of poor performance, was inversely related to their perception that help-seeking is threatening (Karabenick & Gonida 2018). Evidence from this study is consistent with viewing help-seeking in an academic context as achievement-related rather than as dependent behaviours. On the contrary, many students think that asking for academic help from others exposes their academic shortcomings, which can have a negative effect on their self-esteem. If students feel that asking for help may damage their self-esteem, they will be less likely to do so (Elias, Collins & Steiner 2021). One study found that students were more likely to perceive the threat to self-esteem by asking for academic support in person (Reeves 2015).

## Conclusion

This study has demonstrated that some demographic factors, such as gender and marital status, did not influence academic help-seeking behaviour; however, the study identified that home language was significantly associated with academic help-seeking. Students did not see help-seeking as a threat to their self-esteem. The study has shown that most students preferred to use sources such as course materials, books and teachers for academic learning and students also seek academic assistance from different sources.

It is recommended that the college and instructors assist students in seeking help using different useful formal and informal sources, including computer tutorials and YouTube videos for academic learning. There is a need for the establishment of a language support centre for those students struggling with language for academic learning.
